# Efficacy of autologous micrografts technology: a promising approach for chronic wound healing and tissue regeneration—a pilot study

**DOI:** 10.3389/fmed.2024.1417920

**Published:** 2024-07-26

**Authors:** Elisabetta Adelaide Baglioni, Franco Perego, Elisa Paolin, Alberto Abate, Tommaso Pusceddu, Barbara Zavan, Maria Alessandra Bocchiotti

**Affiliations:** ^1^Department of Plastic Surgery, University of Turin, Turin, Italy; ^2^Reconstructive and Aesthetic Plastic Surgery, University of Padua, Padua, Italy; ^3^Human Anatomy Unit, Department of Public Health, Experimental, and Forensic Medicine, University of Pavia, Pavia, Italy; ^4^Laboratory SHRO Italia Foundation ETS, Turin, Italy; ^5^Department of Cardiology, IRCCS San Martino Hospital, University of Genoa, Genoa, Italy; ^6^Department of Translational Medicine, University of Ferrara, Ferrara, Italy; ^7^Department of Plastic Surgeon Gynecological and Obstetrician, City of Health and Science of Turin, Turin, Italy

**Keywords:** micrografts, regenerative medicine, chronic wounds, exosomes, antioxidant activity

## Abstract

**Introduction:**

This study explores the efficacy of Autologous Micrografts Technology (AMG) in treating chronic wounds refractory to traditional therapies.

**Methods:**

AMGs, derived from adipose tissue or dermis using a mechanical fragmentation process, were applied to patients with post-surgical dehiscence. A comprehensive evaluation of wound healing outcomes, including surface area reduction and complete healing, was conducted over a 90-day follow-up period. Additionally, the study investigated the cellular antioxidant activity of AMG solutions and characterized the exosomes obtained through mechanical disaggregation.

**Results:**

Results indicate significant improvements (*p* < 0.05) in wound healing, with 91.66% of patients showing at least a 50% reduction in lesion size and 75% achieving complete healing by day 90. Notably, AMG technology demonstrated immediate efficacy with fat-only application, while combined dermis and fat micrografts showed longer-term benefits, particularly in chronic wounds. The study also elucidated the mechanism of action of AMGs, highlighting their role in enhancing cellular antioxidant activity and exosome-mediated tissue regeneration.

**Discussion:**

Overall, these findings underscore the promising potential of AMG technology as a versatile and effective treatment option for chronic wounds, warranting further investigation into its mechanisms and clinical applications.

## 1 Introduction

The human skin is regarded as the largest organ of the body, accounting for approximately 16% of the adult body weight and covering an area of about two square meters when stretched out. Its histological structure comprises three main layers, namely the epidermis, dermis, and hypodermis, which is also commonly referred to as the subcutaneous fat layer (1).

The dermis is situated directly underneath the epidermis and is responsible for receiving the amplified blood supply to the skin. The majority of skin appendages such as apocrine glands, eccrine glands, and hair follicles are located in the dermis. Comprising of collagen and elastic fibers, the dermis is classified as a connective tissue. The cellular composition of the dermis includes fibroblasts, macrophages, and adipocytes. Additionally, the dermis has two layers, namely the superficial or papillary layer and the deep dermis or reticulate layer (2).

Adipose tissue is a highly active and dynamic tissue composed of various types of cells, including adipocytes, fibroblasts, smooth muscle cells, endothelial cells, and adipogenic progenitor cells (APCs) (3). These preadipocytes, as they are also called, allow for the regeneration of damaged tissues by means of their paracrine, immunomodulatory, chemotactic, and differentiating effects.

APCs belong to the stromal vascular fraction (SVF) of adipose tissue, which also comprises a diverse population of other cell types, such as endothelial cells, pericytes, hematopoietic lineage cells, and fibroblasts (4). The regenerative properties of SVF can be mainly attributed to its paracrine effects. Under stimuli like hypoxia, SVF cells secrete growth factors like vascular endothelial growth factor (VEGF), hepatocyte growth factor (HGF), transforming growth factor beta (TGF-β), and others, which influence stem cell differentiation, promote angiogenesis, and wound healing (5).

APCs have the ability to differentiate into various types of mesodermal tissue and exhibit an expression of similar surface markers as bone marrow-derived cells (6). Cytometric analysis has shown that APCs do not express CD31 and CD45, which are typically expressed by hematopoietic stem cells (7, 8). However, they express CD34, CD73, CD105, CD117, and the mesenchymal stem cell marker CD90 (9). These cells have a differentiation potential similar to other mesenchymal stem cells and a higher rate of proliferation in culture than stem cells derived from bone marrow. Moreover, APCs can be easily obtained without the need for expansion in culture through a standard liposuction procedure under local anesthesia (10).

Surgical and chronic wounds, which are not limited to those persisting for more than 12 weeks (11), but often extend to much longer durations, spanning several years, have a significant socio-economic impact as they affect a large number of patients. Non-healing wounds have various consequences such as an increase in mortality, prolongation of hospitalizations, the need for further hospitalization and re-intervention, delay of adjuvant treatments for other pathologies, impairment of the aesthetic result, and psychosocial wellbeing (12).

The management of such wounds is economically burdensome, with increased direct and indirect costs compared to patients not carrying chronic injuries. Direct costs include frequent changes of medication, management of complications, such as infections, and hospital readmission, while indirect costs consist of the loss of the patient’s income and a decline in socio-benefits. Therefore, accelerating the healing of such wounds would save considerable resources and improve the psycho-social wellbeing of the affected individual and their family members (13).

The Rigenera^®^ (Human Brain Wave, Turin, Italy) autologous micrografts technology (AMG) addresses these issues. It is based on tissue micrografts harvested from the same patient who will receive the micrografting (14). Micrografts from AMG contain progenitor cells embedded within their own extracellular matrix and enriched by various tissue factors, such as cytokines and growth factors, which further increase their regenerative potential (15, 16). This technology has been used for various tissue lesions, such as chronic and dehiscent wounds, ulcers, and burns (17–19). Clinical results and experimental research data confirm the potential of this technology to stimulate the repair of skin tissue through a genuine regenerative process (20, 21).

The aim of this study is to evaluate the effectiveness of the Rigenera^®^ technology in treating chronic post-surgical dehiscence by using micrografts harvested from adipose tissue or a combination of adipose tissue and dermis (22–24).

Dehiscence represents a failure of the surgical procedure, leading to prolonged healing times, numerous hospital visits for dressing changes or re-operations, increased risk of infection in any inserted prosthetic material, extended convalescence, and prolonged absence from work and social activities. In the treatment of dehiscence, it is assumed that the patient was operated on under the best possible conditions and that all risk factors for delayed healing have been corrected. However, even if the surgery was performed optimally, the presence of underlying conditions can lead to chronic wounds that no longer have the ability to heal on their own. Similarly, trophic ulcers of vascular or diabetic origin are always chronic wounds, and their healing is primarily dependent on addressing and correcting the underlying conditions.

Additionally, an in-depth exploration of cellular antioxidant activity using ARPE-19 cells, elucidating the underlying mechanisms contributing to the regenerative potential of AMG, is integrated into the study.

In addition to exploring antioxidant activity, another mechanism of action of the micrografts, namely their exosome-mediated action, was also evaluated. Exosomes represent extracellular vesicles derived from parent cells, encapsulating various biomolecules reflective of their cellular origin. Their versatile nature renders them highly promising in both biotechnological and biomedical domains, serving as prospective indicators of disease states and offering potential therapeutic avenues (25).

These findings shed light on the multifaceted regenerative mechanisms underlying the efficacy of the described micrograft-based treatment.

## 2 Materials and methods

### 2.1 Autologous micrografts technology

**Figure 1:**
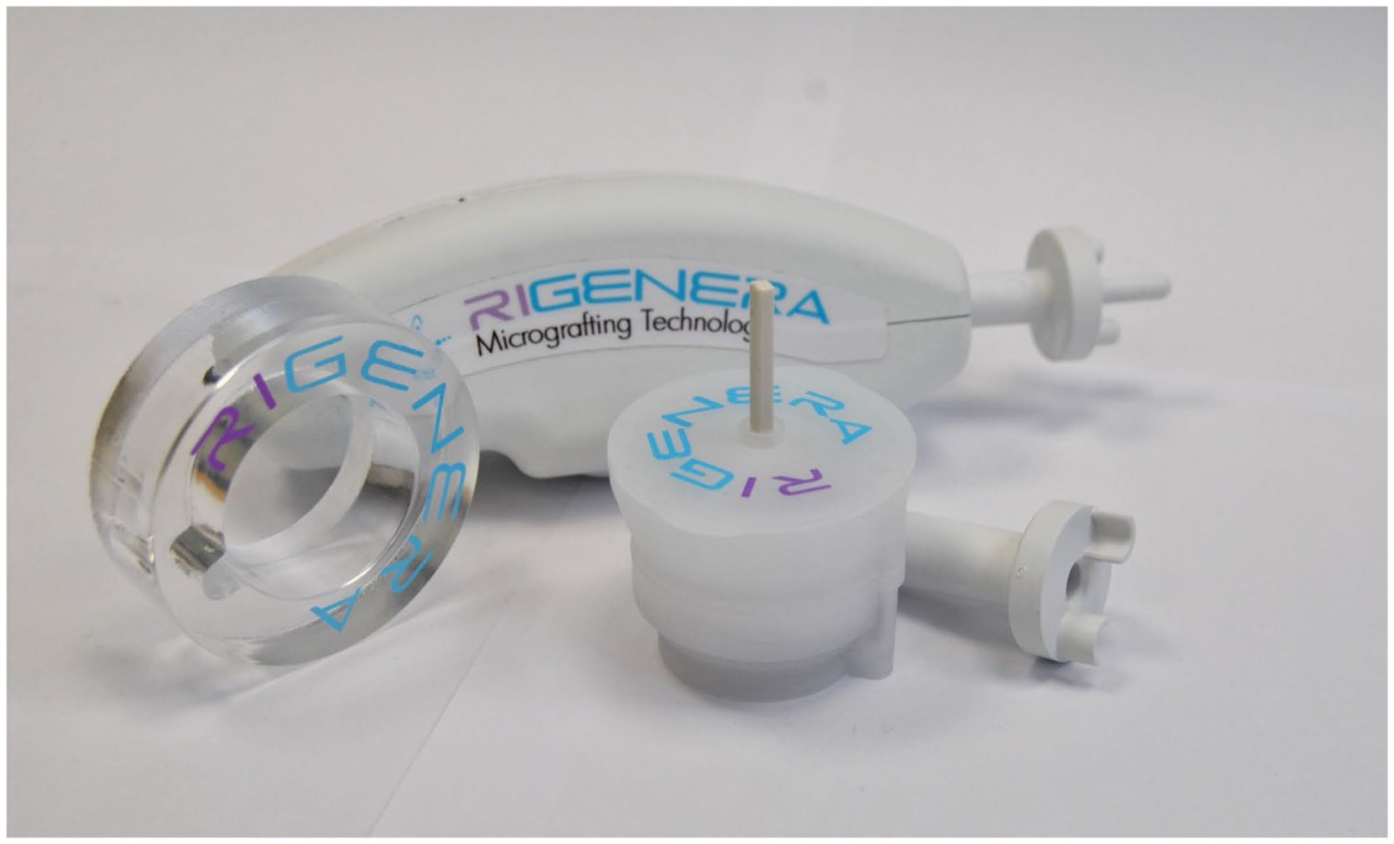
Class II medical device (Rigeneracons) and its electrical motor (Sicurdrill).

The technology is based on the evidence that every solid tissue contains a side-population of progenitor cells that promote tissue regeneration. AMGs were obtained using a Class II medical device consisting of a grid with six micro-blades surrounding hundreds of 80 μm diameter holes, allowing for the selection of AMGs with precise sizes through a dimensional exclusion system. The mechanical fragmentation is activated by an electric motor called Sicurdrill (Figure 1). This technology can break down various tissues, both hard and soft, such as dermis, cartilage, bone, adipose, and cardiac tissue. In this study, fat and dermis were used, with the former processed on its own and the latter requiring the addition of a physiological solution. The AMG solution can be infiltrated along the edges and bottom of a wound or used to imbibe a dermal substitute.

The AMG technology does not require the use of enzymes or additional chemicals, resulting in a procedure that takes only 30 min to complete. This technology stands out by necessitating only a single application, unlike other methods that may require multiple applications. It also eliminates the need for two healthcare operators, as one is sufficient. Importantly, the procedure can be performed without prior scheduling. Lastly, the patient acts as both the tissue donor and the AMG recipient (15, 19, 23, 26–33).

### 2.2 Study design

The study was an uncontrolled experimental investigation that served as a second-line experimental treatment for patients who met the inclusion criteria and had not achieved a significant reduction in wound size from traditional treatments. The traditional treatments employed previously on patients included hydrogel, hydrofiber with or without silver, metalloproteinase inhibitor, silver dressings, polyhexamethylene biguanide (PHMB) dressings, collagen pads, collagenase, hyaluronic acid, and polyurethane foams. A case–control study was not feasible due to the high likelihood of bias between patients and their wounds without appropriate randomization.

Moreover, while a case–control study was possible, it faced critical issues in patient selection. The effectiveness of Rigenera in patients under 80 years without skin diseases is already known. Additionally, the regenerative properties of the stromal vascular fraction of adipose tissue have been known for a long time. This study aims to evaluate the difference in effectiveness of the Rigenera system in selecting adipose tissue APCs by working with small volumes of fat and to compare this method with the combined use of fat and dermis (1–4). The obtained solutions were used in various wounds in patients with and without comorbidities, who are non-responders or have insufficient responses to advanced dressings and sometimes NPWT.

The patients were divided into two groups: the first group received only AMGs using fat, while the second group utilized a combination of dermis and fat. The final analysis aimed to identify any differences between the two groups.

The study was conducted at the A.O.U. Città della Salute e della Scienza in Turin, Italy, at the Department of Plastic Surgery from November 2019 to December 2021. Patients were selected based on specific eligibility criteria, as follows:

Criteria for inclusion:

- Adult patients over the age of 16;
- Post-surgical dehiscence of any origin, present for over 90 days and showing no tendency to healing with advanced dressings, in patients at high risk of alteration of the healing of the surgical wound according to the risk factors exposed in the document of WUWHS: Surgical wound dehiscence 2018 (23);
- Presence or absence of exposure of noble tissues (grade 2, 3, or 4) of depth of post-surgical dehiscence according to WUWHS criteria (23, 25);
- Area < 35 cm^2^.

Criteria for exclusion:

- Lesion >35 cm^2^;
- Large display of noble fabrics for which a flap cover is indicated;
- Wounds other than post-surgical dehiscence;
- Previous negative pressure wound therapy (NPWT) in previous 15 days;
- Previous regenerative medicine in previous 30 days;
- Severe nutritional deficiency, BMI < 18;
- Polychemotherapy in progress;
- Decompensated diabetes;
- Changes in adipose tissue.

### 2.3 Surgical technique

The procedure takes place in an operating room and typically lasts around 30 min. Local anesthesia is administered at the donor sites, with the composition varying depending on whether it is fat or dermis. If the donor site is abdominal or thigh adipose tissue, the composition includes bupivacaine 2% without adrenaline (10 cc), 5 cc bupivacaine 1% with adrenaline, 10 cc physiological solution, and 3 cc sodium bicarbonate. For dermis donor sites, a 5 cc bupivacaine 1% with adrenaline solution is used.

After allowing 5 min for the anesthesia to take effect, a small incision is made with a blade 11 and the adipose tissue is extracted using a 10 cc luer lock syringe connected to a 3 mm diameter Coleman cannula. The lipoaspirate is then washed and decanted with sterile saline solution three times to remove blood, oil, and the local anesthetic.

**Figure 2:**
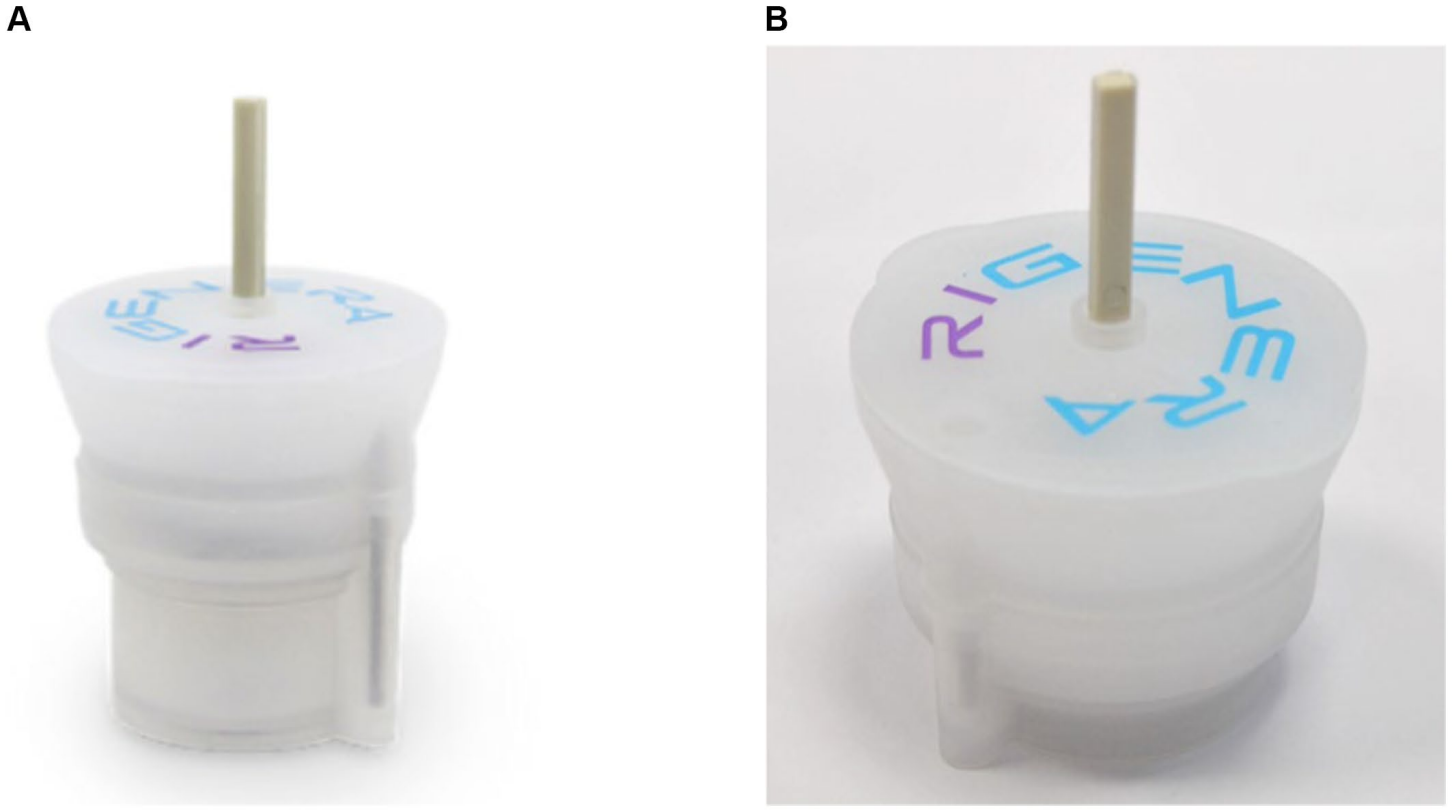
**(A)** Adipecons. **(B)** Rigeneracons TS.

Approximately 10 cc of purified adipose tissue is processed using the Rigenera^®^ device, named Adipecons (Figure 2A), for 3 min at 80 revolutions/min. If a larger amount of AMGs solution is required, the procedure is repeated with further collection of adipose tissue due to the low efficiency of the mechanical breakdown of the adipose tissue (proportion 1:3, 1:4).

AMG solution preparation from dermis involves extraction of a skin fragment 20 times smaller than the lesion using a biopsy punch or scalpel and disintegration of the tissue in the Rigenera^®^ device, called Rigeneracons TS (Figure 2B), by adding 6 mL of physiological solution.

Once the AMGs suspension has been prepared, the lesion is infiltrated using a 5 cc syringe and a 25–27 G needle, with micro-injected drops of solution injected into the deep dermis at a depth of approximately 2 mm and at a distance of 2–4 mm from one another. The needle is held perpendicular to the surface. The solution is also injected into the bottom of the lesion using the same technique.

After the procedure is complete, the remaining solution is used to soak a collagen scaffold, which is placed on the wound as a bioactive dermal substitute. A dressing containing hydrofibre and polyurethane foam is then applied and kept in place for 5 days, with replacement every 3–4 days.

Study was an uncontrolled experimental investigation that served as a second-line experimental treatment for patients who met the inclusion criteria and had not achieved a significant reduction in wound size from traditional treatments. The traditional treatments employed previously on patients included hydrogel matrix, matrix metalloproteinase inhibitors, and polyurethane foams. A case–control study was not feasible due to the high likelihood of bias between patients and their wounds without appropriate randomization.

### 2.4 Post-treatment follow-up

We conducted follow-up evaluations for 90 days with periodic assessments at T0, T7, T30, T40, T60, T70, and T90. During each check-up, the progress of wound healing was documented through photographs and evaluated using a ruler or graph paper, calculation of the surface/volume, and analysis of the bottom, edges, amount, and type of exudate, following the principles of the Wound assessment triangle (WUWHS) (11).

### 2.5 Patients

Fourteen patients were examined in total, but one patient had to be followed up for only 30 days after passing out, and another patient was monitored for 70 days after recently receiving treatment. All patients provided written Informed Consent before undergoing treatment, as required by current regulations. The study was conducted in accordance with the Helsinki Declaration and the European Rules of Good Practice on Cell manufacturing, which include Directives 2004/23/EC and 1394/2007 established by the European Medicines Agency (EMA).

The 14 patients (Table 1) included in the observational study consisted of nine women and five men, aged between 24 and 80 years. The average age was between 50 and 60 years, with a mode of 71–80 years. Four patients did not have comorbidities, while the remaining 10 had multiple pathologies.

**Table 1:** Overview of patient wound characteristics and comorbidities: this table provides a detailed summary of the wound locations, associated comorbidities, types of wounds, wound age, and the use of prosthetic materials in a cohort of patients.

Patient	Wound location	Comorbidities	Type of wound	Wound age	Prosthetic material
1	External malleolus, right leg	Severe obesity, diabetes, heart disease	Traumatic, graft performed about 1 year ago	Over 1 year	None
2	Sacral-coccygeal region	Spina bifida, paraplegia	Pressure ulcer, reopening of wound on previous surgical scar	5 months	None
3	Periumbilical abdominal region	Ovarian tumor, malabsorption syndrome from resection of the small intestine	Surgical wound dehiscence from correctional surgery	5 months	Biological prosthesis (bovine DERMA), exposed
4	Right ischial region	Post-traumatic tetraparesis	Ischial fistula, previous flap coverage	3 months	None
5	Sternal region	Heart disease, COPD, hypertension	Fistula from previous sternal wound revision	8 months	None
6	Left axillary region	Former severe obesity, bariatric surgery, hypothyroidism	Wound dehiscence from mastopexy + arm lift surgery	40 days	None
7	Right forefoot	Diabetes, peripheral vascular disease, ischemic heart disease	Lateral and medial dehiscence from forefoot amputation	8 months	None
8	Scalp	No comorbidities	Chronic ulcerations from previous burns and coverage grafts	1 year	None
9	Left heel	No comorbidities	Trophic ulcer following trauma with ligament lesion	6 months	None
10	Right foot, 3rd toe	No comorbidities	Dorsal dehiscence, 2nd ray right foot from surgical intervention	2 months	None
11	Sternal region	Chronic renal failure, hypertension, Hashimoto’s thyroiditis	Dehiscence with fistula from sternotomy for heart surgery	1 year and 5 months	None
12	Lower abdominal region	Rheumatoid arthritis, chronic steroid therapy	Dehiscence from laparotomy for peritoneal disease	8 months	None
13	Right leg	Hypertension, history of breast cancer under hormone therapy	Traumatic, bimalleolar fracture treated with ORIF	6 months	None
14	Left Achilles region	No comorbidities	Dehiscence from surgical intervention for Achilles tendon repair	8 months	None

The data highlights the diverse nature of wound etiologies and patient health conditions, ranging from traumatic injuries to surgical dehiscences.

The four patients without comorbidities experienced difficulties in wound healing due to the location of the wound (toe, weight-bearing area of the sole, Achilles tendon, and scar tissue on the scalp).

The patients with multiple pathologies had the following comorbidities: two patients had obesity, four had heart disease, two had diabetes, three were on antiplatelet therapy, one had active non-cutaneous cancer, two had chronic obstructive pulmonary disease, one had chronic kidney disease, one had rheumatoid arthritis on steroid therapy, one had peripheral vascular disease, four had hypertension, two had thyroid disorders, one had radiation dermatitis, one had paraplegia, and one had tetraplegia.

Each patient selected at the Plastic Surgery Clinic of the City of Health and Science University Hospital in Turin followed a standardized path before accessing regenerative medicine:

- Collection of clinical history
- Analysis of the wound: dimensions/volume, base, edges, exudate, exposure of noble tissues or prosthetic implants.
- Application of wound bed preparation (WBP) criteria following TIME principles using hydrogel, collagenase, hydrofiber, and PU foam. Antiseptic dressings were used if there were signs of infection: PHMb gauze, ionized silver gauze, cadexomer iodine.
- Once the wound bed was prepared, bioactive dressings promoting re-epithelialization, such as collagen and hyaluronic acid, were applied.

Only after at least 60 days of therapy following these principles, with little or no success, did the patients proceed to regenerative medicine to reactivate the wound healing processes that were not responding to other methods.

### 2.6 Data and statistical analysis

The data were collected using electronic data collection forms (e-CRF), which were developed by the A.O.U. Epidemiological and Evaluation SSD - CPO of the Città della Salute and della Scienza of Turin, and captured through the Electronic Data Capture system. ANOVA test and linear regression were employed to analyze the collected data.

### 2.7 Quantification of cellular antioxidant activity of AMG solution: a comprehensive assay using ARPE-19 cells and Trolox equivalents

ARPE-19 cells were seeded in 96-well microplates and allowed to adhere for 24 h. Subsequently, they were incubated for 48 h with the AMG solution. In the development of this assay, cells were maintained in non-supplemented DMEM-F12, and control wells were included without the means of study. The following solutions were utilized:

- AMG solution
- Braun physiological serum 0.9% (B. Braun Medical, SA)
- Commercial medium, DMEM-F12 supplemented with 10% FCS

After 2 days, the culture medium was removed, and a pretreatment was conducted, involving the addition of Trolox [(±)-6-hydroxy-2,5,7,8-tetramethyl-chroman-2- carboxylic acid], a water-soluble synthetic analog of vitamin E, as an antioxidant at standard concentrations between 0 and 2000 μM. Simultaneously, cells were incubated with 2′,7′-diacetate dichlorodihydrofluorescein (DCFH-DA), a fluorescent probe permeable to cells retained inside the cell by the action of intracellular esterases. This treatment lasted for 60 min at 37°C, after which the cells were washed and dried.

Following this, the cells were incubated with 200 μL of PBS, and a free radical generator, 2,2′-Azobis(2-methylpropionamidine) dihydrochloride (AAPH), was added. Immediately after adding AAPH, a fluorescence kinetics study was conducted with readings every 2 min for 1 h, maintaining the plate at 37°C. Readings were taken at an excitation wavelength of 485 nm and an emission wavelength of 525 nm. DCFH-DA was rapidly oxidized by free radicals to 2′,7′-Dichlorodihydrofluorescein diacetate (DCFH), a highly fluorescent compound.

The assay aimed to measure cellular antioxidant capacity in preventing the formation of DCF by peroxyl radicals, generated by AAPH in cells. A decrease in cellular fluorescence compared to control cells indicated the antioxidant capacity of the compounds (Figure 1A). Results were expressed in Cellular Antioxidant Activity (CAA) and in micromolar (μM) of Trolox equivalents (TE). Each plate included triplicates of Trolox for each concentration and whites (presence of the probe, but not oxidant). All other determinations were performed in triplicate from five independent experiments.

At the end of the time reading, cell viability was assessed using the MTT method. Cellular Antioxidant Activity was quantified by the Integration Area under the Curve (AUC) normalized to the number of live cells (MTT) in each well. To process the obtained data, the results of each sample were interpolated with the Trolox standard curve [CAA units vs. (Trolox, μM)] to obtain the equivalents of Trolox, using GraphPad Prism 9.2 software (GraphPad Software Inc., San Diego, CA, United States).

### 2.8 Rigenera^®^ micrografts solution: mechanism of action exosomes mediated

The primary objective of the study is to characterize the population of extracellular nanovesicles, known as exosomes, obtained from mechanically disaggregated skin biopsies utilizing Rigenera^®^ devices. The investigation focused on biopsies obtained from both cranio-facial (FC) and abdominal (AD) areas. Following each disaggregation, a suspension of the disaggregated product was generated, and extracellular nanovesicles were subsequently isolated. These nanovesicles underwent quantitative characterization through protein analysis and qualitative assessment via Nanoparticle Tracking Analysis (NTA), providing insights into vesicle diameter and distribution. Additionally, cytometric analysis was employed to scrutinize surface markers. Activity analysis involved gene expression analysis of markers associated with tissue regeneration, such as growth factors and metalloproteinases, using inflamed *in vitro* human dermal fibroblasts (HDF). To supplement these analyses, scanning electron microscopic images were acquired for a more comprehensive understanding.

#### 2.8.1 Mechanical disaggregation of biopsies

Skin biopsies, obtained using 3 mm diameter skin biopsy punches and immersed in saline solution for preservation, were disaggregated using Rigeneracons devices. Each biopsy sample from a single donor was processed individually (Table 2). The biopsies were washed with sterile saline solution and placed on the grid of the disaggregation device. Through the designated hole, 2 mL of sterile saline solution were inoculated using a syringe without a needle. The device was positioned in the anchor base, and the electronic part of the equipment guiding the disaggregation process was fixed on top. Two consecutive disaggregation cycles of 1 min each were performed for each sample. At the end of the disaggregation process, the suspension of the disaggregated product containing all the disaggregation products was extracted using a needle-free sterile syringe and transferred into a sterile tube.

**Table 2:** The table lists the processed samples and the devices used.

Device	ID	Biopsy	Donor
Rigeneracons	AD1	Abdominal skin	Gender F—Age 87
Rigeneracons	FC1	Cranio-facial skin	Gender M—Age 38
Rigeneracons	FC2	Cranio-facial skin	Gender M—Age 46
Rigeneracons	FC3	Cranio-facial skin	Gender M—Age 26
Rigeneracons	AD2	Abdominal skin	Gender M—Age 61
Rigeneracons	FC4	Cranio-facial skin	Gender M—Age 45
Rigeneracons	FC5	Cranio-facial skin	Gender M—Age 23

#### 2.8.2 Isolation of exosomes

The suspension of disaggregated micrografts obtained was subjected to centrifugation at 1200 rpm for 4 min to sediment cellular debris. Following centrifugation, the supernatant was collected and filtered using a 0.22 μm filter. Exosomes were subsequently isolated from the filtrate through ultrafiltration.

#### 2.8.3 Quantitative and dimensional characterization

The exosome population underwent quantitative and dimensional analysis utilizing Nanoparticle tracking analysis (NTA) conducted with NanoSight (Malvern Panalytical, Malvern, United Kingdom). This analysis provided information on the average size and concentration of the vesicle populations.

#### 2.8.4 Characterization of surface markers

The confirmation of exosomes within the obtained vesicle suspension was achieved through flow cytometric analysis, which identified specific exosomal surface markers including CD81, CD63, and CD9 (34). The flow cytometric analysis was conducted using the Attune NxT Acoustic Focusing Cytometer (Life Technologies, Carlsbad, CA, United States), and the resulting data were analyzed using Attune NxT version 2.5 software (Life Technologies).

#### 2.8.5 Culture and treatment of human dermal fibroblasts

Human dermal fibroblasts (ATTC, Manassas, Virginia) were cultured in Dulbecco’s Modified Eagle’s Medium (DMEM, Euroclone, Milan, Italy), supplemented with 10% Fetal Bovine Serum (Euroclone) and 2% Penicillin–Streptomycin (Sigma), at 37°C, 5% CO_2_ in a humidified environment until reaching confluence. The experimental procedure involved seeding 0.05^*^106 cells per well in a 24-well plate. After 48 h of seeding, an inflammatory treatment was administered; the fibroblasts were incubated for 24 h with inflammatory DMEM medium (Euroclone) supplemented with 10 ng/mL TNFα (Merck, Darmstadt, Germany). Following the inflammation period, the medium was replaced with fresh culture medium, and the cells were treated with the previously isolated exosome suspensions, followed by an additional 24 h of incubation.

#### 2.8.6 Extraction and purification of cellular RNA

At the conclusion of the incubation period, cell lysis was performed, and total RNA extraction was carried out using Total RNA Purification Kits (Norgen Biotek Corp, Thorold, ON, Canada), following the manufacturer’s provided protocol.

#### 2.8.7 Gene expression analysis

The quantification of RNA samples was conducted using NanoDrop (Thermo Fisher Scientific, Waltham, MA, United States). Subsequently, reverse transcription was performed utilizing the SensiFAST cDNA Synthesis Kit (Meridian Bioscience, Cincinnati, OH, United States). Gene expression analysis was carried out using Rotor-Gene Q (Qiagen, Hilden, Germany) and SensiFAST™ SYBR^®^ No-ROX Kit (Meridian Bioscience), following the manufacturers protocol. The acquired data were then processed using Q-Rex Software (Qiagen).

Genes involved in the study were IL10, FGF2, VEGFA, TGFB1, IL1B, TNF, MMP1, MMP9, COL1A1, COL3A1 (Table 3).

**Table 3:** Sequences of primers for the genes examined in the study.

Genes	Gene names	Primer direction	Primer sequences	Annealing temperature (°C)	Amplicon size (bp)
TGFβ1	Transforming growth factor, beta 1	F	TGACAGCAGGGATAACACACT	63.9	194
		R	CCGTGGAGCTGAAGCA	64	
VEGFα	Vascular endothelial growth factor A	F	GGACAGAAAGACAGATCACAGGTAC	64.3	182
		R	GCAGGTGAGAGTAAGCGAAGG	64.9	
MMP1	Matrix metallopeptidase 1	F	TCACACCTCTGACATTCACCAA	64.2	183
		R	TGGTCCACCTTTCATCTTCATCA	63.9	
MMP9	Matrix metallopeptidase 9	F	TCTGGAGGTTCGACGTGA	63.9	183
		R	GGTCCACCTGGTTCAACTCA	64	
FGF2	Fibroblast growth factor 2 (basic)	F	TCTTTCAGCATTCACACCACTACA	64.9	153
		R	GCCAACTCGTAACAATCCATCAGAA	64.4	
COL1A1	Collagen, type I, alpha 1	F	GGTTCGGAGGAGAGTCAGGAA	64.9	143
		R	ACGAGGTAGTCTTTCAGCAACAC	64.4	
COL3A1	Collagen, type III, alpha 1	F	AAAGACGCATGTTATGGTGCTA	64.6	124
		R	GCCAATGAATCTTTCTGATGGGAGAC	65.3	
TNF	Tumor necrosis factor	F	GAGGGAGAGAAGCAACTACAGA	63.3	145
		R	AGTGCTCATGGTGTCCTTTCC	64.3	
IL1β	Interleukin 1, beta	F	TCATTCGCTCCCACATTCTGA	64.8	177
		R	AAAGAGAGCACACCAGTCCAA	65	
IL10	Interleukin 10	F	TCTCCGAGATGCCTTCAGC	64.3	193
		R	CGCCTTGATGTCTGGGTCTTG	65.2	

## 3 Results

### 3.1 Clinical results

Out of the total patients, 9 of them achieved complete healing by T90, with 4 of them reaching it by T70 and 2 by T30 (Table 4). Notably, 2 of these patients had multiple injuries, and one of them achieved complete healing in some of the wounds. The remaining 5 patients saw varied results, with only 3 reaching T90, of which 2 recorded a reduction in lesion size of at least 50% by T90, while the other did not reach the 50% threshold even by T90.

**Table 4:** The table contains the data analyzed for the study, with measurements recorded in cm^2^ for each of the specified time points (T0, T7, T30, T40, T60, T70, and T90).

N°	Gender	Procedure	Comorbidities	T0 WS	T7 WS	T30 WS	T40 WS	T60 WS	T70 WS	T90 WS
1	M	F + D	+	3.75	2.00	2.30	1.50	2.50	2.50	2.50
2	F	F	+	3.75	3.75	0.49	PF	PF	PF	PF
3	F	F + D	+	0.80	0.36	0.09	NA	NA	NA	0
4	M	F + D	+	12.00	0.09	0.04	NA	0.01	0.01	0
5	M	F + D	+	0.16	0.16	0.09	0.04	0.01	0.01	0
6	F	F	+	1.5	0.5	0	0	0	0	0
7A	M	F + D	+	1.84	1.84	0.68	NA	0.50	0	0
7B				4.00	4.00	2.30	NA	1.95	0.7	0
8A	F	F	−	9.00	9.00	1	0.16	0.10	0.10	0.16
8B				1.00	1.00	0.40	0	0	0	0
8C				0.16	0.16	0.04	0	0	0	0
8D				0.16	0.16	0.04	0	0	0	0
9	M	F + D	−	1.96	1.44	0.64	0.36	0.09	0	0
10	F	F + D	−	1.00	1.00	0.04	0	0	0	0
11	F	F	+	0.09	0.09	0	0	0	0	0
12	F	F	+	1.75	1.75	0	0	0	0	0
13	F	F + D	+	9.75	9.75	5.50	4.00	2.10	1.20	0.60
14	F	F + D	−	3.75	3.75	3.12	NA	2.55	FU	FU

The legend for the table is as follows: F, fat technique; F + D, fat plus dermis; PF, represents cases lost on follow-up; NA, not applicable; FU, cases still in follow-up; WS, wound size expressed in centimeters.

Therefore, in 91.66% of cases, there was a notable improvement in lesion size of at least 50% among the 12 patients who reached T90, with 75% of them achieving complete healing. Currently, the data is being further analyzed based on gender, procedure, and comorbidity.

Furthermore, a collection of images depicting patient progress and lesion improvement accompany this analysis, providing visual insights into the diverse outcomes observed in the study.

**Figure 3:**
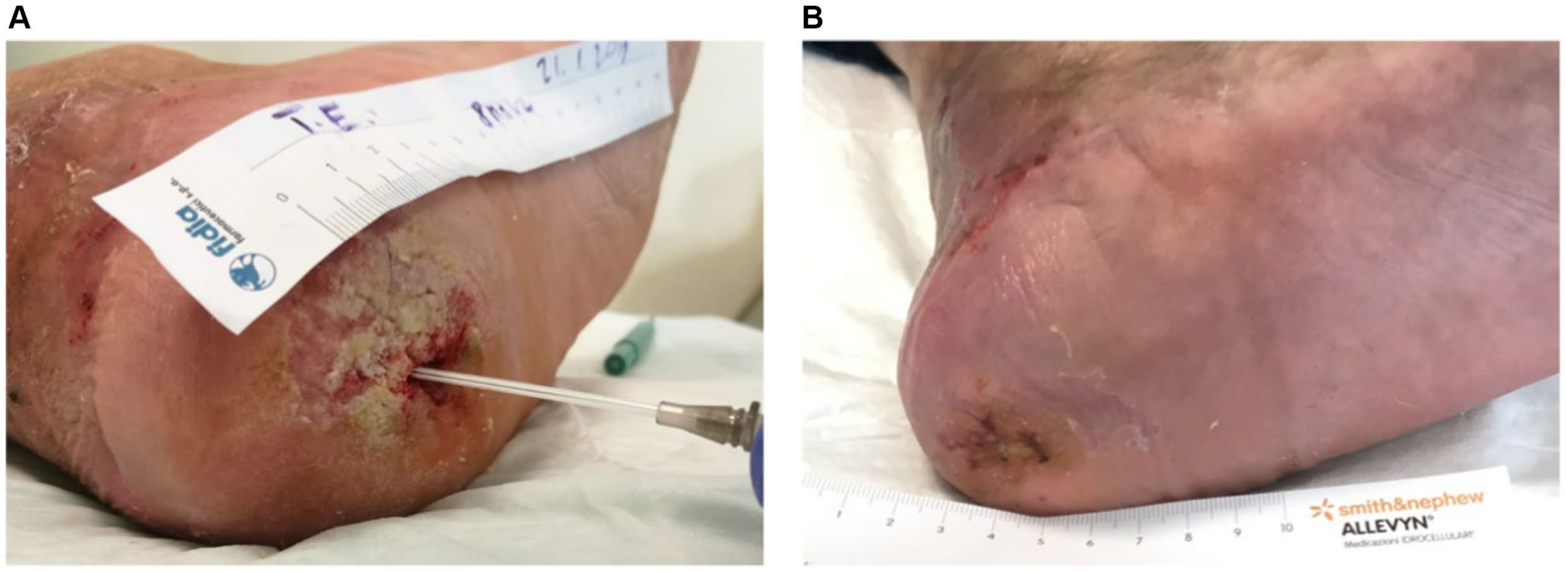
**(A)** The image depicts the treatment progress in a 42-year-old patient with a trophic heel ulcer resulting from a post-traumatic injury to the external popliteal sciatic nerve. **(B)** Despite lasting over a year and being unresponsive to standard care, complete healing was achieved in approximately 60 days with Rigenera^®^ dermis and fat treatment.

For instance, Figure 3 illustrates the treatment progress in a 42-year-old patient with no comorbidities presented with a trophic ulcer on the heel resulting from a post-traumatic injury to the external popliteal sciatic nerve (SPE), leading to reduced sensitivity in the heel. The ulcer persisted for over a year and showed no response to standard advanced wound care treatments. The patient underwent Rigenera^®^ dermis and fat treatment, resulting in complete healing in approximately 60 days.

**Figure 4:**
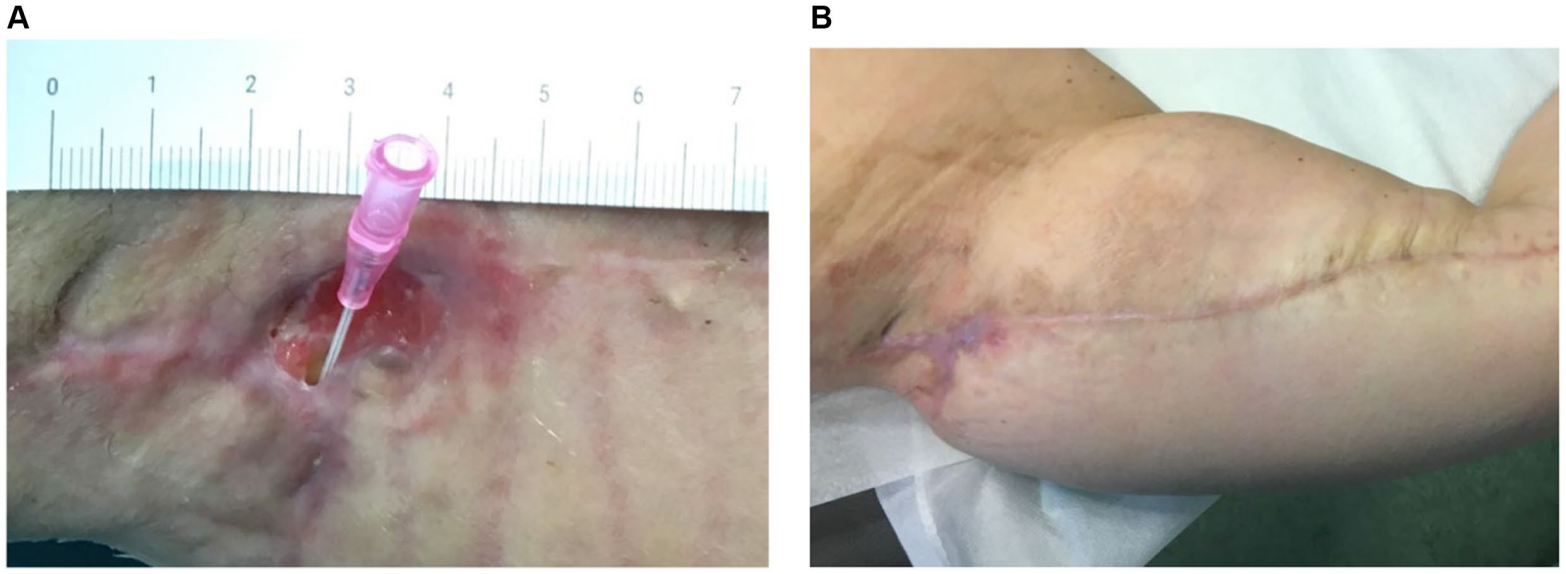
**(A)** The image depicts the treatment progress in a 52-year-old female, previously morbidly obese, underwent successful bariatric surgery, losing 40 kg. Following mastopexy and chest lifting, she experienced non-responsive wound dehiscence in the left axilla for over 30 days. **(B)** Rigenera^®^ dermis+fat treatment resulted in complete healing in about 20 days.

Figure 4 illustrates the treatment progress in a 52-year-old female patient, formerly morbidly obese, underwent a bariatric surgery resulting in a weight loss of 40 kg. She subsequently underwent mastopexy with chest lifting. A non-responsive wound dehiscence in the left axilla persisted for over 30 days despite standard wound care. The patient received Rigenera^®^ dermis and fat treatment and achieved complete healing in approximately 20 days.

**Figure 5:**
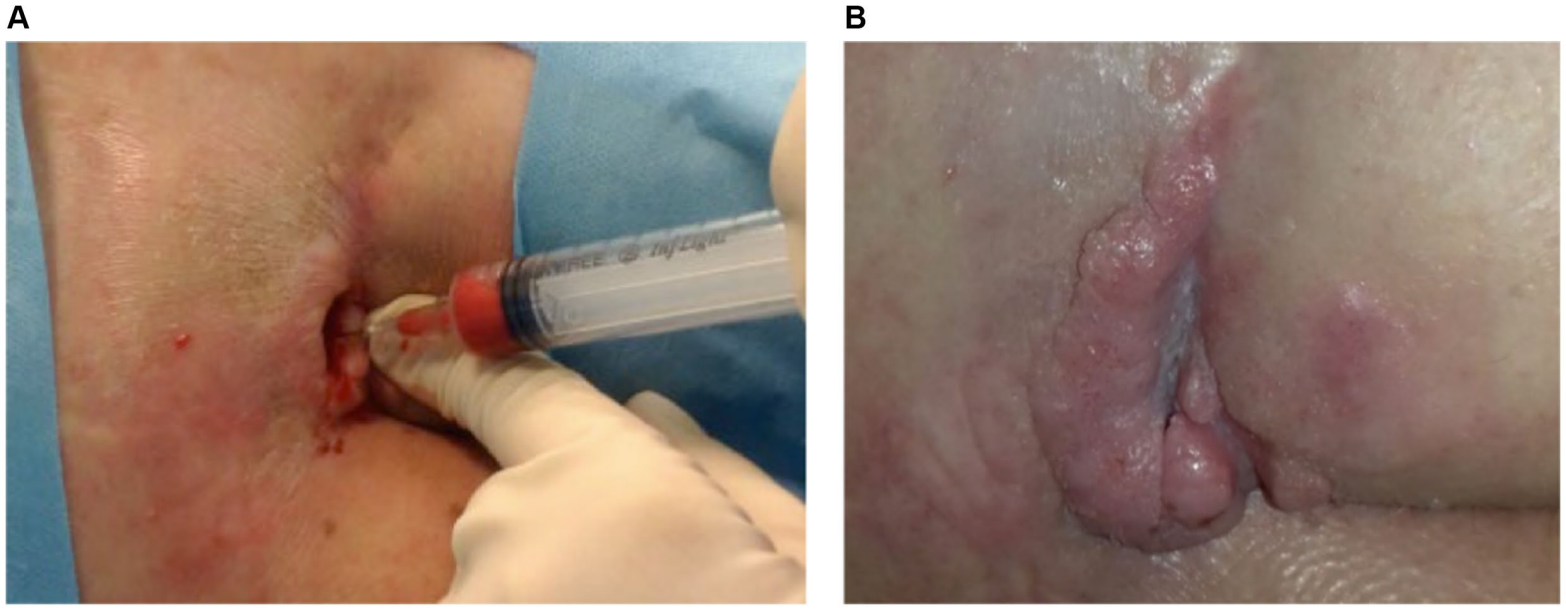
**(A)** A 22-year-old with spina bifida and diabetes had a persistent sacral ulcer post-spinal surgery for over 6 months. While advanced wound care initially helped, progress stopped. **(B)** Rigenera^®^ dermis+fat treatment led to complete healing in 160 days.

Figure 5 illustrates the treatment progress in a 22-year-old girl with spina bifida and diabetes presented with a sacral pressure ulcer in the aftermath of spinal stabilization surgery, persisting for over 6 months. Initial improvement was achieved with advanced wound care, but progress halted. Rigenera^®^ dermis+fat treatment was administered, resulting in complete healing in 160 days.

### 3.2 Procedure and micrograft type impact outcomes over time: a group-based analysis of fat-only and fat and dermis micrografts

The data analysis revealed that at T30, 50% of the healing process was achieved in 83.3% of patients treated with only fat, while the success rate was 56.25% for those treated with the combined dermis and fat approach.

By T70, the goal of 100% healing was reached by the fat technique alone in 55% of the patients, with a success rate of 41.66% for the combined technique.

Notably, at T90, both techniques showed an increase in the number of patients achieving complete healing, with a success rate of 85.71% for the dermis-fat approach and 75% for the fat technique.

These findings suggest that the dermis-fat approach may have longer-term beneficial effects that manifest with a certain delay, while the fat technique has more immediate effects that are sustained in the short term.

### 3.3 Procedure evaluation considering comorbidities

After 30 days of treatment, 83.33% of patients without comorbidities achieved complete healing, in contrast to 56.25% of patients with comorbidities. These findings suggest that patients without additional pathologies are more likely to exhibit a favorable response to therapy within the initial 30 days.

At T70, 75% of patients without comorbidities reached the endpoint, demonstrating relatively stable progress compared to T30. In contrast, among patients with comorbidities, only 25% achieved 100% healing. This underscores the significant impact of comorbidities on the wound healing process. Patients without pre-existing pathologies affecting tissue healing respond more favorably than their counterparts with comorbidities.

To gain deeper insights into the influence of different pathologies on tissue regeneration, it is recommended to categorize and classify them systematically. This approach would enable a standardized study based on comorbidities, facilitating a comprehensive evaluation of the distinct impact of each pathology and potentially enhancing the overall healing process.

### 3.4 Cellular antioxidant activity

**Figure 6:**
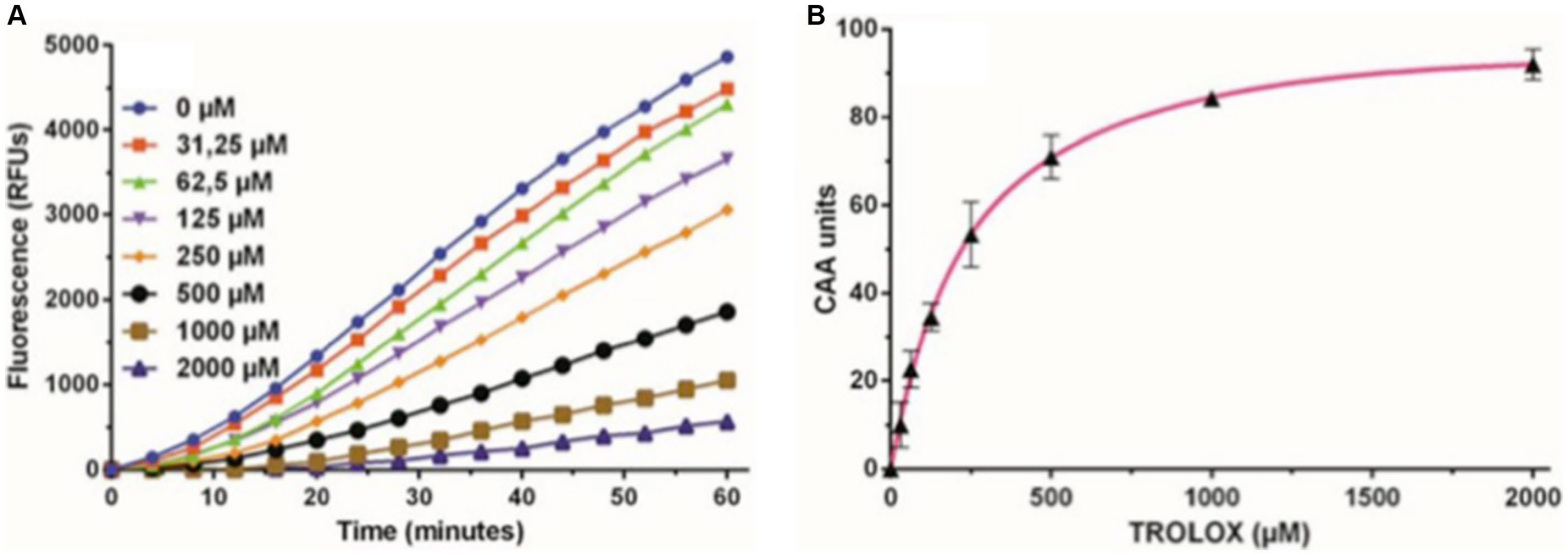
Trolox kinetics graphs. **(A)** Representative fluorescence curves depicting the Cellular Antioxidant Activity (CAA) assay using different concentrations of the antioxidant Trolox as a standard, ranging from 0 to 2000 μM. **(B)** Dose–response curve of the Trolox standard, illustrating the net area under the curve (AUC) for various concentrations of Trolox antioxidant standards, ranging from 0 to 2000 μM. These values were used to calculate CAA units plotted against Trolox concentration. The subsequent calibration curve facilitated the interpolation of Trolox equivalents (TE) for the tested compounds.

In assessing intracellular free radical production, ARPE-19 cells were treated with the apolar and nonfluorescent compound 2′,7′-dichlorodihydrofluorescein diacetate (DCFH-DA). Following deacetylation by cytosolic esterases, DCFH-DA transforms into dichlorodihydrofluorescein (DCFH), a polar non-fluorescent molecule. DCFH reacts with free radicals (ROS), yielding the fluorescent derivative dichlorofluorescein. The cells were cultured in a monolayer within a 96-well microplate for 48 h in the three mentioned incubation media. Trolox, at various concentrations, served as an antioxidant reference (Figure 6). AAPH free radicals were then added to initiate the assay, with fluorescence readings taken every 2 min for 1 h at 37°C. The resulting fluorescence kinetics, reflecting DCF formation, were monitored for 1 h. Cellular Antioxidant Activity (CAA) was determined as the reduction in the percentage of intracellular free radicals compared to the antioxidant-free control, expressed as Trolox equivalents (TE) concentration (Figure 6A), and quantified by the Integration Area Under the Curve (AUC) (Figure 6B).

In the control culture medium, basal cellular antioxidant activity remained consistently below 10 μM Trolox equivalents (Table 5). Saline addition maintained a lower basal cellular antioxidant activity (5–10 μM of Trolox, Table 5). The inclusion of AMG significantly enhanced cellular antioxidant capacity (*p* < 0.05) across all doses compared to saline (1.5–6 times) and the culture medium (2–6 times) (Table 5). This protective effect exhibited peak efficiency at a 25% volume, decreasing by 50% at higher values, a trend consistent with cell viability and proliferation studies.

**Table 5:** Comparative effect of cellular antioxidant capacity (CAA) at 48 h of treatment (mean ± SD) in healthy ARPE-19 cells.

Antioxidant activity 48 h		% final volume					
		2.50%	5%	10%	15%	25%	50%
Culture media	Trolox equivalent (μM)	3.23	0.29	3.61	7.48	6.16	6.95
	±	1.23	0.32	0.63	2.35	1.56	1.54
	n	3	3	3	3	3	3
Saline solution	Trolox equivalent (μM)	5.43	7.15	6.95	9.32	6.61	6.19
	±	3.71	1.19	2.30	4.13	0.47	0.89
	n	3	3	3	3	3	3
AMG solution	Trolox equivalent (μM)	8.30	9.19	12.11	24.48	37.18	24.73
	±	3.74	4.24	0.69	3.79	9.10	8.37
	n	3	3	3	3	3	3

**Figure 7:**
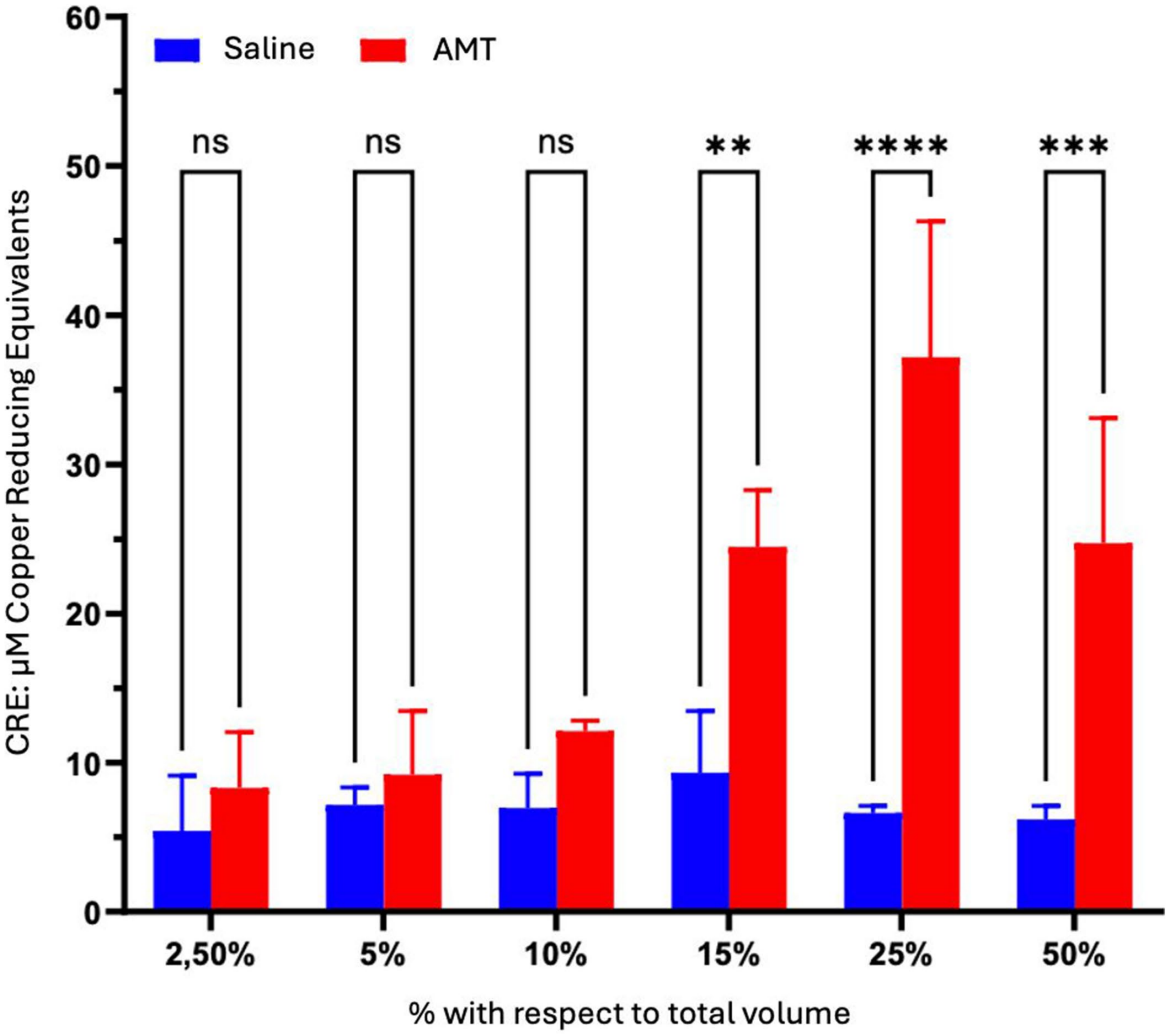
Comparison of the impact of AMT dosage on the Cellular Antioxidant Capacity (CAA) in ARPE-19 cells after 48 h of treatment. The cells were exposed to culture medium containing varying proportions of saline or AMT over the specified duration. Viability was assessed through the MTT assay. Intergroup statistical analysis was conducted using a two-way ANOVA test followed by a Bonferroni post-test. ^*^p < 0.05; ^**^p < 0.01; ^***^p < 0.001; ^****^p < 0.0001.

Statistically significant differences (*p* < 0.05) between saline and AMG extract were observed at concentrations exceeding 10% by volume, demonstrating maximum effectiveness at 25%. At this point, antioxidant activity was equivalent to a Trolox concentration of approximately 40 μM (Figure 7).

### 3.5 Characterization of exosomes

**Figure 8:**
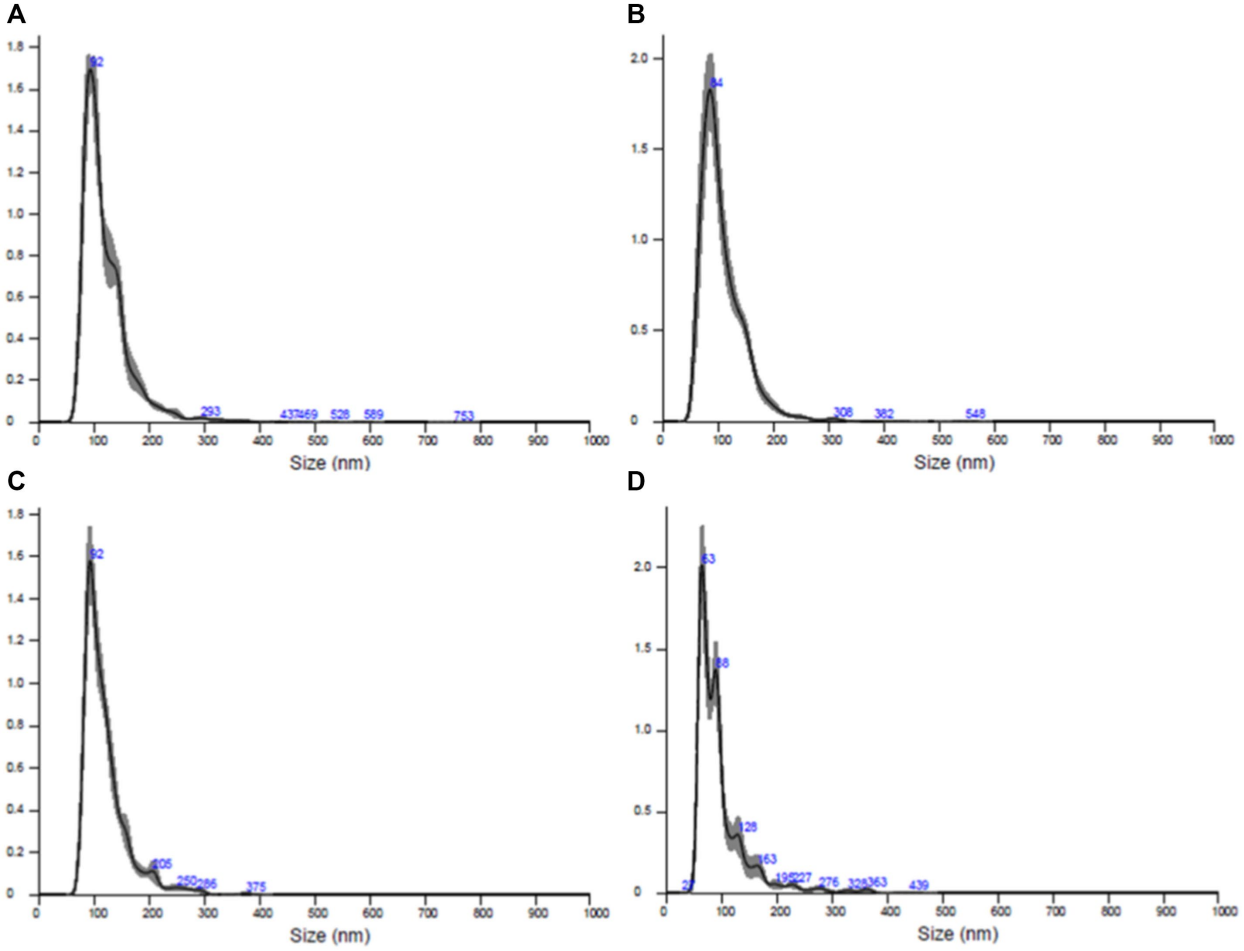
Results of the quantitative and dimensional analysis of exosome populations isolated from skin samples conducted using nanoparticle tracking analysis (NTA). **(A)** Sample AD1, **(B)** sample FC1, **(C)** sample FC2, **(D)** sample FC3.

**Figure 9:**
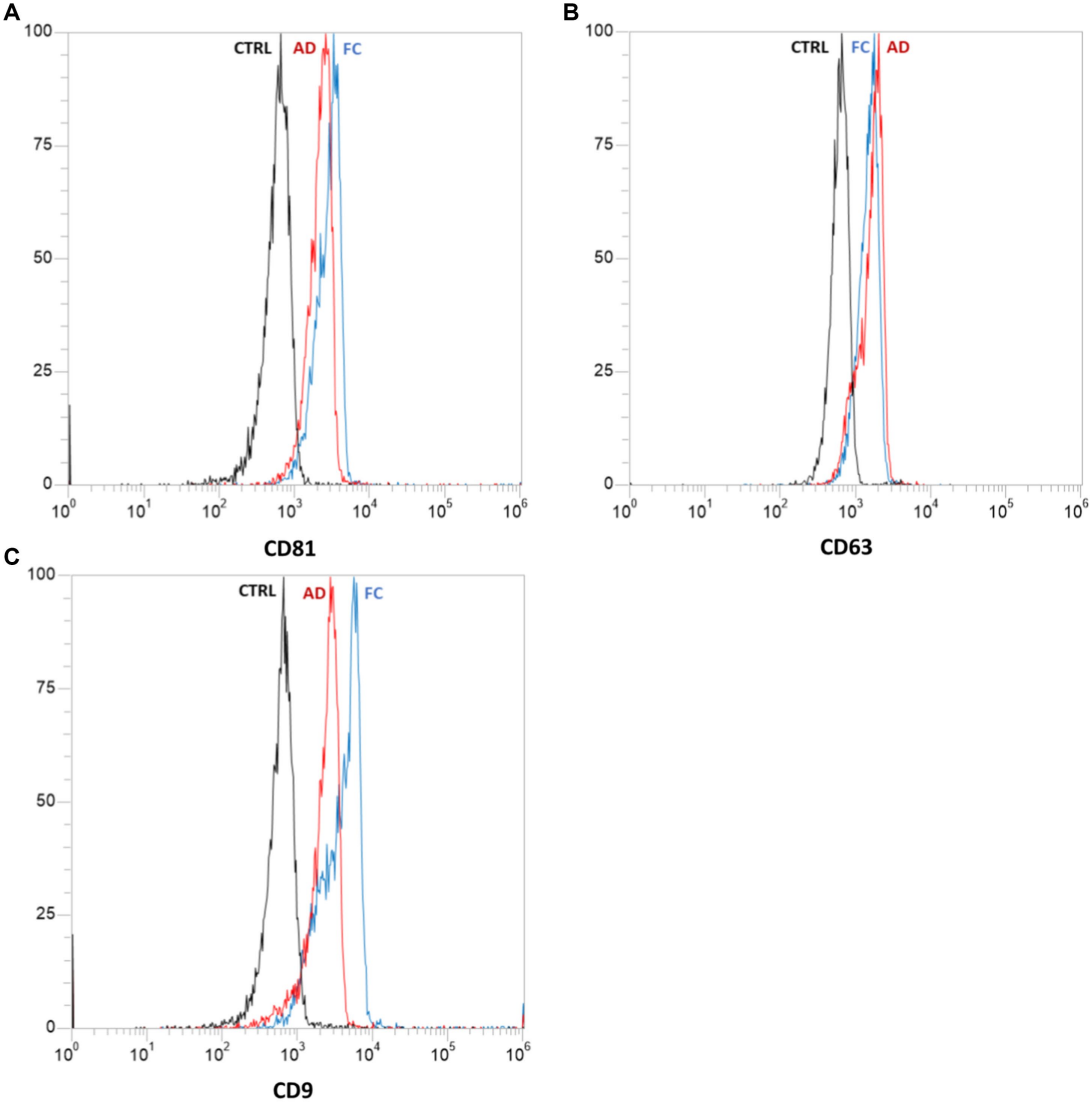
Results of flow cytometric analysis of specific exosomal surface markers: CD81 **(A)**, CD63 **(B)**, and CD9 **(C)**. Each graph represents the negative control “CTRL” (in black) and samples derived from abdominal skin biopsies “AD” (in red) and cranio-facial skin biopsies “FC” (in blue). Both samples tested positive for the presence of all three surface markers.

All samples underwent processing, and the vesicles were subjected to analysis. The characterization of extracellular vescicoles unveiled the existence of exosome populations, showcasing an average diameter spanning from 95.9 nm to 119.5 nm and a concentration falling within the range of 108–1,010 particles/ml (Figure 8). The populations of extracellular nanovesicles exhibited positive outcomes for the presence of exosome-specific surface markers (Figure 9). It is noteworthy that both Figures 8, 9 present results solely for a subset of the involved samples, not encompassing the entirety of the dataset.

The exosomes derived from cranio-facial and abdominal skin samples were utilized for the treatment of inflamed human fibroblasts. The collected data were then normalized against the untreated inflamed control. Gene expression analysis revealed a distinct expression pattern of genes associated with wound healing and regeneration in cell populations treated with exosomes compared to the control (Table 6).

**Table 6:** Results of the differential gene expression analysis performed by RT-qPCR.

Gene		Fold change	
		AD	FC1
TNF	Tumor necrosis factor	1.08	1.08
IL1B	Interleukin 1 beta	1.95	2.69
COL1A1	Collagen 1 A III	0.71	0.65
COL3A1	Collagen 3 A I	2.34	3.39
FGF2	Fibroblast growth factor 2	5.92	8.91
VEGFA	Vascular endothelial growth factor	1.98	2.68
IL10	Interleukin 10	1.97	6.70
TGFB1	Transforming growth factor beta 1	0.75	0.74
MMP1	Matrix metalloproteinase 1	1.89	2.21
MMP9	Matrix metalloproteinase 9	2.93	7.19

**Figure 10:**
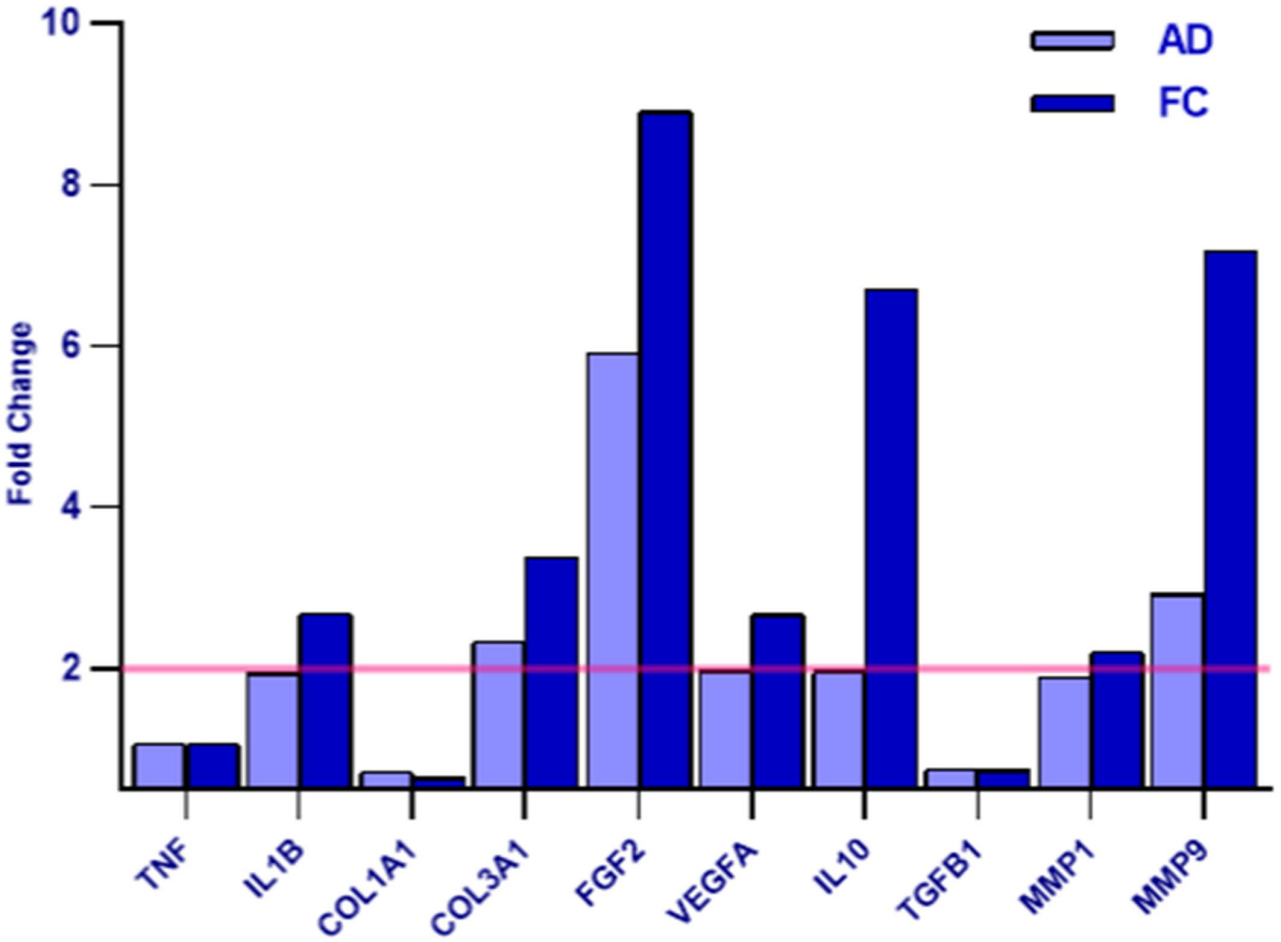
The graph depicts the AD and FC samples - as indicated in the legend - on the x-axis and the differential expression indicated as the Fold Change value on the y-axis. A Fold Change value ≥2 indicates an increase in the expression of the examined gene in the sample compared to the control (highlighted in yellow), while a value <2 indicates that no difference in expression was detected between the two conditions.

The variance in gene expression between cranio-facial and abdominal skin samples is quantified as the fold change, with a value ≥2 indicating an increased expression of the examined gene in the sample relative to the control, and a value <2 indicating no significant difference in expression between the two conditions (Figure 10).

## 4 Discussion

The study followed up on 14 patients with a total of 18 wounds’ sites. Among them, two patients did not complete the study. Of the 12 patients who reached T90, nine wounds had 100% full recovery, four by T70 and two by T30.

Overall, the study found that 91.66% of the 12 patients who reached T90 showed an improvement on the surface of the lesion of at least 50%, while 75% showed complete healing. This suggests that the method is promising for patients who are not responding to other therapies or advanced treatments for chronic wounds.

**Figure 11:**
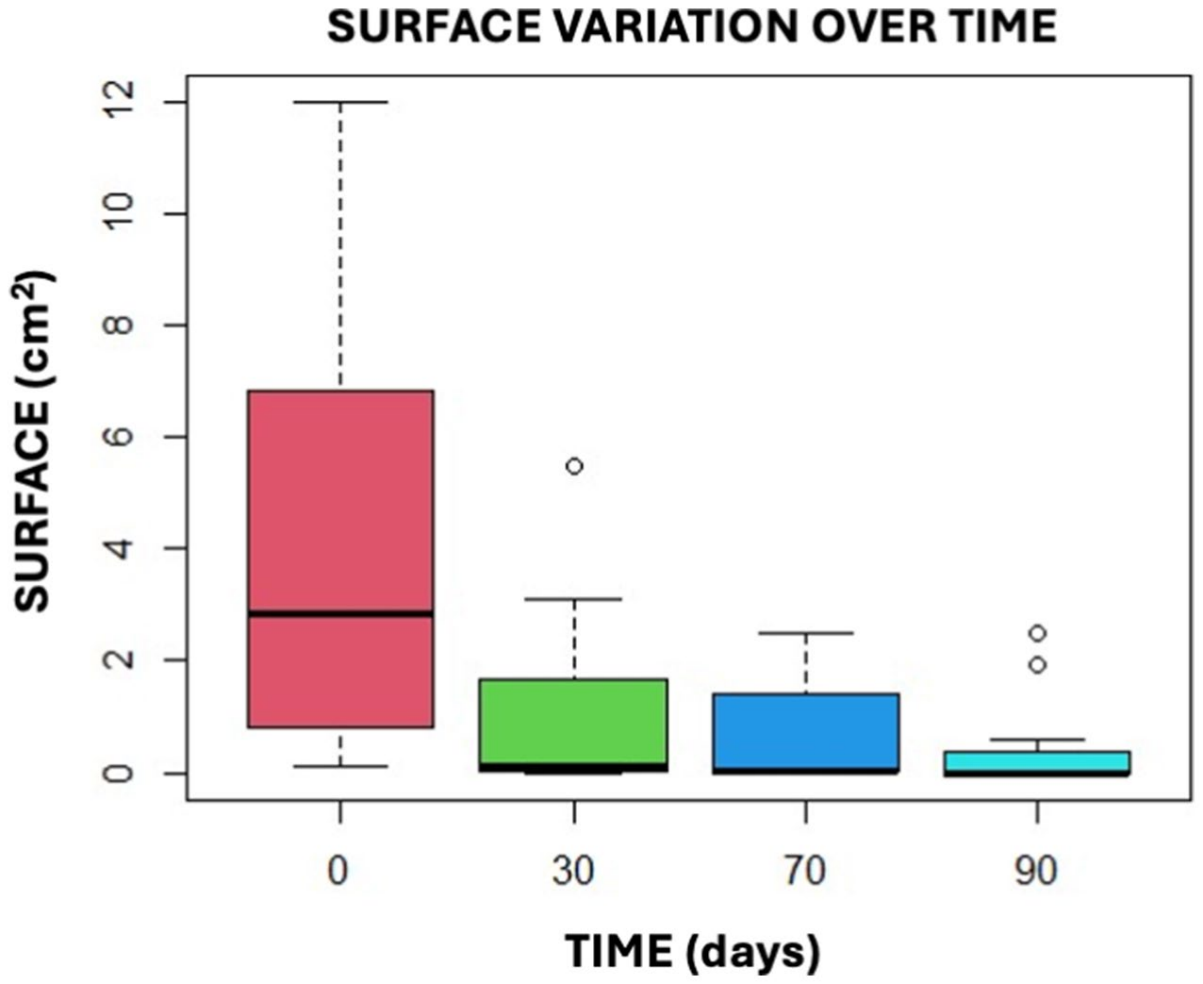
This graphical representation illustrates the findings of the Anova Test by presenting the data in the form of confidence intervals.

The study suggests that the reduction in surface area between T0 and T30, T70, and T90 is significant, as shown in the box plot graphs provided (Figure 11).

The study also identified several advantages of the technique, including the ability to be carried out on an outpatient basis and with no need for other structures or instruments for sample processing. Additionally, it is essential to note that this is a one-shot technology and the results are observable from the first application, as demonstrated by the data presented in the study, supported by previous research findings (14–24). The technique can be performed on any type of patient, including those with extreme comorbidities, and has no side effects for the patient.

Recalling the purpose of the study, which was to compare the use of Rigenera^®^ technology with the use of fat alone and the combination of dermis and fat, the observation of the patients included in the study revealed that fat exhibits a more immediate impact within 30 days, while the dermis exerts its effects over the long term. Regarding sub-acute wounds, using only fat or only dermis proves effective. However, for chronic wounds, a combination of dermis and fat is recommended due to their synergistic effect.

Furthermore, in cases where dermal use is contraindicated due to dermal pathologies, it is established that fat alone can be employed, making it a versatile option applicable to all patients, including those with conditions like scleroderma or skin tumors. Notably, it is unsuitable for conditions such as keratosis or *in situ* tumors.

Despite the effectiveness of fat in providing an initial push toward wound healing, and its viability for reinforcement when dermal application is preferred, it is essential to emphasize that micrografts harvested from the dermis remain the optimal choice in wound treatment. Nevertheless, when dermal use is not feasible, fat alone remains a viable option.

Another distinctive advantage of the technology used lies in the fact that through it, it is possible to use small volumes of fat, enabling the treatment of small wounds on any patient type, even in those with chronic use of anti-aggregation drugs or in those who have skin problems. However, when dealing with larger wounds, a greater amount of material is necessary, leading to the exclusion of certain patient profiles, such as individuals with very low body weight.

Collectively, both previous studies and the present investigation indicate the promising potential of the technique in treating chronic wounds. In addition to the existing evidences on the technology, a new avenue for future research has emerged. This involves the observation that fat demonstrates efficacy in achieving short-term results, whereas the dermis exhibits the capacity for long-term outcomes. Therefore, the combination of dermis and fat appears to be an advantageous choice. However, this warrants further exploration and understanding, necessitating an expanded study with a larger cohort to delve into the intricacies of this dermis-fat pairing for enhanced comprehension in the future.

A hypothesized mechanism, yet to be verified, is that fat coupled with dermis may act as a sort of natural scaffold, aiding in filling the cavity of the wound. This potential scaffold function of the fat-dermis pairing could play a crucial role in facilitating wound healing processes. Further investigation is essential to confirm this hypothesis and elucidate the underlying mechanisms behind the observed therapeutic effects.

The study, although conducted on a small sample of patients, has demonstrated the effectiveness of both methods: isolated fat and the combination of fat and dermis. The combined use of fat and dermis has proven to be an optimal solution for all types of chronic wounds such as wound dehiscence, as it is an easy and feasible technique for small wounds in every kind of patient. Comorbidities seem to be non-significant for wound healing following Rigenera^®^ treatment, but it is also true that comorbidities could delay wound healing until 90 days after treatment. It is also important to remember that the efficacy of the Rigenera^®^ technology for the treatment of wounds using dermis alone is well known, but in elderly patients, it is less efficient. The use of both dermis and fat together could improve the efficacy of this method because we have demonstrated in this study the early efficacy of APCs of adipose tissue in tissue regeneration in elderly people as well. Another advantage is that the Rigenera^®^ adipose solution is in a fluid form, allowing injection into wound edges and the wound floor with very low pain and no need for analgesic drugs. As we have worked with people with many comorbidities, which means the assumption of a lot of drugs, this is an advantage.

Certainly, the small number of samples does not allow us to express a firm and sure opinion about the effectiveness and reproducibility of these results; however, the data obtained are encouraging and open the way to future studies on larger samples. It is important to note that one significant limitation of this study is the lack of a control group. The absence of a control group makes it difficult to draw definitive conclusions about the superiority of the Rigenera^®^ technology compared to other treatments. Future studies should incorporate a control group to provide more robust evidence of the efficacy and superiority of this treatment. Such studies would benefit from randomization and larger sample sizes to validate these preliminary findings and ensure that the results are both reproducible and generalizable.

In parallel, the study integrates a multi-faceted understanding behind the Rigenera^®^ Technology. In particular, findings related to cellular antioxidant activity induced by the AMG solution have emerged, as well as an exosome-mediated mechanism. The significant enhancement in antioxidant capacity, observed through comparative analysis with both complete culture medium and saline control, underscores the potential therapeutic implications of the AMG solution in mitigating oxidative stress. It is crucial for wound care technology to include an antioxidant mechanism. Chronic wounds often have high levels of free radicals, disrupting the body’s defenses and healing process. Antioxidants can help restore balance, speeding up recovery and lowering complication risks. They also protect surrounding tissues, reducing damage and infection risks (35). The intricate interplay between AMG components and cellular antioxidant pathways remains an intriguing aspect, urging further exploration to unravel the underlying mechanisms.

Additionally, the discovery that the Rigenera^®^ solution contains exosomes further reinforces its therapeutic efficacy. Exosomes, extracellular nanovesicles, hold considerable significance in regenerative medicine. These vesicles are continuously secreted by various cell types, with their content and biological functions closely tied to the biological state of the progenitor cell. Characterized by dimensions ranging from 30 to 200 nm in diameter and marked by specific surface indicators such as CD81, CD63, and CD91, these nanovesicles play a pivotal role in cellular communication.

Analysis of extracellular nanovesicles isolated in this study confirmed that the Rigeneracons device effectively isolates exosomes, measuring approximately 150 nanometers, and exhibiting specific surface markers consistent with literature reports.

The disintegration of skin biopsies using Rigeneracons devices enables the creation of a suspension containing cutaneous-derived exosomes. These exosomes demonstrated the capability to modulate the gene expression of inflamed human dermal fibroblasts, influencing specific genes associated with tissue regeneration mechanisms, matrix remodeling, angiogenesis, and cell proliferation.

Notably, the analysis did not reveal a significant difference in the effect of exosomes derived from abdominal and cranio-facial biopsies, except for the higher expression of the anti-inflammatory IL10 in facial samples. However, this disparity was not considered significant, given the considerably higher age (87) of the patient in the abdominal sample compared to the average age of donors for facial region samples (average age 40).

The study identified that treatment with Rigenera^®^ exosomes led to an increase in the synthesis of various factors, including IL10 (an inhibitor of pro-inflammatory cytokine synthesis) (34), FGF2 (a factor stimulating cellular proliferation and tissue repair) (36), VEGFA (promoting angiogenesis in wound healing) (37), MMP9 (involved in angiogenesis, wound closure, and matrix remodeling) (38), COL3A1 (a protein contributing to the structural integrity of many tissues), IL1B (an inflammation mediator), and MMP16 (involved in extracellular matrix remodeling) (39). No significant differential expression was observed for TGFB1 (an inhibitor of cytokine secretion), TNFa (a pro-inflammatory factor), and COL1A1 (a structural protein present in many tissues).

## 5 Conclusion

In conclusion, the results of the study indicate that the combined derma-fat approach exhibit favorable outcomes both in the short and long term, while fat alone provides benefits primarily in the short term. Moreover, derma, whose efficacy has been widely demonstrated by previous studies published in the literature, is capable of exerting long-term effects compared to fat. This finding highlights the importance of considering the timeline of effects when selecting a suitable micrograft technique, and underscores the need for a personalized approach that takes into account the unique needs and preferences of each patient.

Moreover, the technique has many advantages, which can be summarized in its simplicity of execution, need of only one operator to be executed, single application, outpatient execution and possibility to be extended to almost all categories of patients.

The collective findings support the technology’s promise in treating chronic wounds, offering both short-term efficacy and the prospect of long-term outcomes, especially when combining dermal and fat micrografts. This integrated perspective paves the way for future research directions, emphasizing the need for larger cohorts and in-depth molecular investigations to solidify the clinical applicability of the Rigenera^®^ technology and the therapeutic implications of the AMG solution.

The concluding segment of the study aimed to explore additional mechanisms of action of Rigenera^®^ micrografts. Specifically, the goal was to determine if, alongside the presence of progenitor cells, extracellular matrix, and inherent growth factors in the micrograft solution, there was also an antioxidant mechanism of action and an exosomes-mediated mechanism of action. In conclusion, this comprehensive understanding sheds light on the multifaceted therapeutic potential of Rigenera^®^ micrografts, demonstrating their efficacy not only in younger patients but also in the elderly. Additionally, the study affirms that Rigenera^®^ technology is suitable for treating wounds of any type, from superficial to deep, and of varying sizes. Moreover, the treatment of chronic wounds appears to be more effective when the synergistic combination of dermis and fat is used.

Furthermore, it highlights the discovery of two additional mechanisms of action, encompassing both exosome-mediated and antioxidant mechanisms.

It is important to acknowledge that the absence of a control group in this study is a limitation. Future studies should include a control group to provide more robust evidence and ensure that the observed effects can be attributed to the treatment itself rather than other variables. Such studies will be crucial for validating the promising results observed and for establishing a clearer understanding of the efficacy of the Rigenera^®^ technology.

## Data availability statement

The datasets presented in this study can be found in online repositories. The names of the repository/repositories and accession number(s) can be found in the article/supplementary material.

## Ethics statement

The studies involving humans were approved by A.O.U. Città della Salute e della Scienza—University of Turin. The studies were conducted in accordance with the local legislation and institutional requirements. The participants provided their written informed consent to participate in this study. Written informed consent was obtained from the individual(s) for the publication of any potentially identifiable images or data included in this article.

## Author contributions

EB: Conceptualization, Investigation, Methodology, Project administration, Supervision, Writing – original draft. FP: Investigation, Methodology, Resources, Writing – review & editing. EP: Conceptualization, Data curation, Investigation, Validation, Writing – review & editing. AA: Conceptualization, Data curation, Formal analysis, Writing – review & editing. TP: Data curation, Formal analysis, Validation, Writing – review & editing. BZ: Conceptualization, Formal analysis, Methodology, Supervision, Validation, Writing – review & editing. MB: Conceptualization, Investigation, Methodology, Supervision, Visualization, Writing – review & editing.
